# Endermic Use of Tobacco

**Published:** 1845-09

**Authors:** Wm. Beck Diver

**Affiliations:** Cincinnati


*Endermic use of Tobacco.* By Wm. Beck Diver, M. D., of Cincinnati.

[Communicated by Professor Dunglison.]

Cincinnati, Aug. 5th, 1845.

*Dear Sir,*—I send herewith a copy of the 11th number of the Western Lancet, containing the results of some experiments on the "endermic use of tobacco," which you may esteem worthy of being communicated still further to the profession.

The efficacy of this plan in "Hysteria," and "Spasmodic Stricture of the Urethra," has been fully set forth in the Lancet, vol. 2, page 420, which you have doubtless observed.

I take the liberty of communicating these facts to you, presuming upon your superior ability and opportunity to make them more generally known.

With the "endermic use of iodine" my success in scrofula has been not less encouraging.

In four well marked cases I succeeded in discussing active scrofulous swellings of the lymphatic glands of the neck, by the use of the following formula, suggested by Mr. Charles Smith, an able pharmacien of Philadelphia.

℞. Iodin. p. j.
Balsam. Canad., p. iij.
Picis abietis, p. iij.

The iodine to be triturated with the balsam: the pitch to be melted with a gentle heat, and, as it is about to cool, the whole to be mixed and spread upon kid for immediate use. We thus avoid the volatilization of the iodine, and at the same time have a neat and convenient form of external application, free from the objections which apply to the iodine ointment.

In cases where the extreme sensitiveness of the parts precludes the use of inunction, I should give a decided preference to this method; not relying exclusively, however, upon the endermic use of the remedy. * * * * *

Yours, most truly,
Wm. Beck Diver.

Professor R. Dunglison.

The following is the article in the Western Lancet, referred to by Dr. Diver.

*Endermic use of Tobacco.* By Wm. Beck Diver, M. D., of Cincinnati.—The use of Tobacco as a remedial agent has received less attention from the profession than it deserves. Its powerful agency has been satisfactorily tested in cases of strangulated hernia, ileus, and spasmodic stricture of the urethra; but the dangerous consequences which have been reported as resulting from its use in the usual form by the mouth and by the rectum, induced me some years ago to try it endermically; and the results, which were entirely satisfactory, were published in the Lancet, Vol. II, page 420.

Subsequent trials of this powerful antispasmodic lead me to repose still greater confidence in it, and induce me strongly to recommend it in cases where the ordinary remedies of this class fail or are inadmissible.

The following case will illustrate the advantages of this method in tetanic rigidity of the muscles.

W. H., of St. Charles, Mo., received, about seven years ago, a wound from an adze, which completely divided the rectus femoris muscle immediately above the patella. In order to procure healing by "the first intention," his physician, very properly, placed the limb in the extended position; his object was attained, but subsequent inflammation and deposition in the knee joint produced anchylosis, and the most unyielding rigidity of the limb. Up to the time, June, 1844, when the patient was submitted to my care, he had travelled about with great difficulty, and derived no advantage from medical treatment. He was extremely anxious to regain, if possible, the use of his knee joint, and therefore cheerfully submitted to an operation for this purpose. In the presence of several physicians of this city, I divided the rectus femoris transversely in nearly the direction of the original wound; this was effected with a very small knife by a subcutaneous incision; scarcely a drop of blood was effused; the ends of the divided muscle widely retracted, and a very slight degree of flexion could be produced by the application of force. The next day, 13th June, I placed the limb in Roe's apparatus for compound fracture of the leg, and applied the screw, by which a still greater degree of flexion was produced. For several weeks flexion and extension were kept up, until a good degree of motion was established in the knee joint. Beyond a certain point, however, the antagonist muscles refused to yield, and upon applying a greater degree of force with the screw, a severe tetanic rigidity was set up in the extensors which threatened to frustrate our object. To relieve this I directed the patient, who was unaccustomed to the use of tobacco, to smoke freely the strongest that could be procured. While under the influence of this form of the remedy, the muscles became relaxed, and a still further flexion of the knee was effected. The following ointment was rubbed into the groins and over the knee:

℞. Iodin. gr. xij.

Potassæ Hydriod. ℈iv.

Olei Nicotianæ, gtt. L.

Adipis Præparat. ℥ij. Misce.

Under a persevering use of this formula, and continual application of the screw producing flexion and extension, I succeeded in reducing the tetanic rigidity of the muscles and tendons; the motion of the joint was in a good degree restored, and the patient enabled to ride on horseback with perfect satisfaction. His health being slightly impaired by the confinement necessary, he was advised to visit Kentucky, where he rode and walked with more ease and pleasure than he had enjoyed for many years before.

I have succeeded in relieving a violent cerebral and gastric irritation by the powerful antiphlogistic properties of this agent; and the results of my experience in the endermic use of tobacco are such as to lead me to prefer it in cases where antiphlogistic means are requisite, but where the vital powers are too much depressed to admit of venesection, or the internal use of antimonials. The application of tobacco cataplasm to the epigastrium has, in my practice, removed violent colic resulting from spasmodic stricture of a portion of the intestinal tube, and copious evacuations have been produced by the same method where the ordinary purgatives were inadmissible, or when delay was dangerous. A great advantage in this method is that the remedy is more immediately under the control of the practitioner than when administered internally.

